# A meta-analysis of alcohol drinking and cancer risk

**DOI:** 10.1054/bjoc.2001.2140

**Published:** 2001-11

**Authors:** V Bagnardi, M Blangiardo, C La Vecchia, G Corrao

**Affiliations:** Dipartimento di Statistica, Università di Milano-Bicocca, via Bicocca degli Arcimboldi 8, Milano, 20126, Italy; Istituto di Statistica Medica e Biometria, Università degli Studi di Milano, via Venezian 1, Milano, 20133, Italy; , Istituto di Ricerche Farmacologiche “Mario Negri”, via Eritrea 62, Milano, 20157, Italy

**Keywords:** alcohol intake, neoplasms, humans, meta-analysis, risk

## Abstract

To evaluate the strength of the evidence provided by the epidemiological literature on the association between alcohol consumption and the risk of 18 neoplasms, we performed a search of the epidemiological literature from 1966 to 2000 using several bibliographic databases. Meta-regression models were fitted considering linear and non-linear effects of alcohol intake. The effects of characteristics of the studies, of selected covariates (tobacco) and of the gender of individuals included in the studies, were also investigated as putative sources of heterogeneity of the estimates. A total of 235 studies including over 117 000 cases were considered. Strong trends in risk were observed for cancers of the oral cavity and pharynx, oesophagus and larynx. Less strong direct relations were observed for cancers of the stomach, colon and rectum, liver, breast and ovary. For all these diseases, significant increased risks were found also for ethanol intake of 25 g per day. No significant nor consistent relation was observed for cancers of the pancreas, lung, prostate or bladder. Allowance for tobacco appreciably modified the relations with laryngeal, lung and bladder cancers, but not those with oral, oesophageal or colorectal cancers. This meta-analysis showed no evidence of a threshold effect for most alcohol-related neoplasms. The inference is limited by absence of distinction between lifelong abstainers and former drinkers in several studies, and the possible selective inclusion of relevant sites only in cohort studies. © 2001 Cancer Research Campaign http://www.bjcancer.com


British Journal of Cancer (2001) 85(11), 1700–1705
© 2001 Cancer Research Campaign
doi: 10.1054/ bjoc.2001.2140, available online at http://www.idealibrary.com on IDEAL
http://www.bjcancer.com

### A meta-analysis of alcohol drinking and cancer risk

**V Bagnardi[1], M Blangiardo[1], C La Vecchia[2,3] and G Corrao[1]**

[1]Dipartimento di Statistica, Università di Milano-Bicocca, via Bicocca degli Arcimboldi 8, 20126 Milano, Italy; [2]Istituto di Statistica Medica e Biometria, Università degli Studi di Milano, via Venezian 1, 20133 Milano, Italy; [3]Istituto di Ricerche Farmacologiche "Mario Negri", via Eritrea 62, 20157 Milano, Italy

**Summary** To evaluate the strength of the evidence provided by the epidemiological literature on the association between alcohol consumption and the risk of 18 neoplasms, we performed a search of the epidemiological literature from 1966 to 2000 using several bibliographic databases. Meta-regression models were fitted considering linear and non-linear effects of alcohol intake. The effects of characteristics of the studies, of selected covariates (tobacco) and of the gender of individuals included in the studies, were also investigated as putative sources of heterogeneity of the estimates. A total of 235 studies including over 117 000 cases were considered. Strong trends in risk were observed for cancers of the oral cavity and pharynx, oesophagus and larynx. Less strong direct relations were observed for cancers of the stomach, colon and rectum, liver, breast and ovary. For all these diseases, significant increased risks were found also for ethanol intake of 25 g per day. No significant nor consistent relation was observed for cancers of the pancreas, lung, prostate or bladder. Allowance for tobacco appreciably modified the relations with laryngeal, lung and bladder cancers, but not those with oral, oesophageal or colorectal cancers. This meta-analysis showed no evidence of a threshold effect for most alcohol-related neoplasms. The inference is limited by absence of distinction between lifelong abstainers and former drinkers in several studies, and the possible selective inclusion of relevant sites only in cohort studies. © 2001 Cancer Research Campaign http://www.bjcancer.com

**Keywords**: alcohol intake; neoplasms, humans; meta-analysis; risk

Although alcohol is not known to be carcinogenic in animal experimentation, there is strong epidemiological evidence that alcoholic beverages increase the risk of cancers of the oral cavity and pharynx, oesophagus and larynx. The risks are essentially due to total ethanol intake, and tend to increase with the amount of ethanol drunk, but it is still unclear whether there is any threshold below which no effect is evident (IARC, 1988; Doll et al, 1999). Alcohol drinking has been associated with primary liver cancer, although this relation is difficult to investigate in epidemiological studies, since most alcohol-related liver cancers follow a cirrhosis, which leads to a reduction of alcohol drinking (Aricò et al, 1994; La Vecchia et al, 1998).

Alcohol drinking has also been linked to cancers of the large bowel in both sexes and of the female breast. Although these associations are still open to discussion, these are the 2 most common neoplasms in developed countries after lung cancer, and therefore even a moderate excess risk may have important public health implications. The association between alcohol drinking and several other neoplasms, including ovary, endometrium and bladder, is still controversial (IARC, 1988; Doll et al, 1999).

To evaluate the effect of alcohol on cancer risk, a more accurate quantification of its effects on various neoplasms is required. With this major focus, we therefore updated in the present paper a comprehensive meta-analysis of published case–control and cohort studies investigating the possible relation between alcohol intake and the risk of several neoplasm (Corrao et al, 1999). We also addressed the issue of the potential modifying effect of tobacco on alcohol-related cancerogenesis at various cancer sites.

*Received 23 July 2001*
*Revised 7 September 2001*
*Accepted 14 September 2001*

*Correspondence to:* C La Vecchia

### METHODS

The statistical methods used for this meta-analysis are described in detail elsewhere (Corrao et al, 1999, 2000). Articles included were found through a search of the literature published from 1966 to 2000. The search was based on several bibliographic data-bases (MEDLINE, Current Contents, EMBASE, CAB Abstracts and Core Biomedical Collection), supplemented by checking all references in the selected articles. Completeness was verified by a hand-search on the most relevant journals of epidemiology and medicine, and by comparing our search with that of general reviews and meta-analyses published on this issue (IARC, 1988; Longnecker et al, 1990; Longnecker, 1994, 1995; Longnecker and Enger, 1996; Corrao et al, 1999; Doll et al, 1999; Zeegers et al, 1999; Dennis, 2000).

Each publication identified by this process was reviewed and included in the analysis if the following criteria were met: (i) case–control or cohort study published as an original article; (ii) findings expressed as odds ratio or relative risk (RR), considering at least 3 levels of alcohol consumption; (iii) papers reporting the number of cases and non-cases, and the estimates of the odds ratios or RR for each exposure level. Multivariate RRs were used for the main analysis purpose. When the results of a study were published more than once, only the most complete data were included in the analysis.

2 readers, blinded to the authors' names and affiliations and to the results pertaining to alcohol consumption, independently determined the eligibility of each paper. Pooled estimates of the effect of alcohol consumption on the risk of each neoplasm investigated were based on several meta-regression models (Greenland and Longnecker, 1992). Briefly, the data analysis strategy first involved pooling the original data for each neoplasm; subsequently the relationship between alcohol consumption and risk was modelled by fitting several fractional models (Royston et al, 1999) in order to identify J- or U-shaped curves, or other relations between alcohol exposure level and relative risks. In the current application, a family of second-order models was generated by power transformation of exposure variable, and the best-fitting model was chosen to summarise the relation of interest. The effects of gender and of the adjustment of the reported estimates for smoking in modifying the effect of alcohol on the risk of each neoplasm were also investigated by comparing pooled estimates based on RRs unadjusted for tobacco to the adjusted ones used in the main analysis, whenever available (Berlin et al, 1993). Heterogeneity among studies was evaluated according to the method described by Greenland and Longnecker (1992).

### RESULTS

Altogether, 235 studies with a total of 117 471 cases, met the inclusion criteria and were considered in the analysis. Of the included studies, 187 were case–control and 48 cohort studies, investigating the risk of 18 cancer sites, or of all cancers irrespective of site.

The main characteristics and results of the studies, based on multivariate analysis, are given in Table 1. For 5 cancer sites (melanoma and cancer of small intestine, gallbladder, cervix uteri and kidney) the estimates were based on one or 2 studies only, and did not consider the effects of high alcohol consumption.

Strong direct trends in risk were observed for cancers of the oral cavity and pharynx (RR = 6.0 for 100 g day$^{-1}$), oesophagus (RR = 4.2) and larynx (RR = 3.9). Direct relations – though appreciably less strong – were also found for cancers of the stomach (RR = 1.32), colo-rectum (RR = 1.38) and liver (RR = 1.86), as well as for breast (RR = 1.7 for 50 g day$^{-1}$ and 2.7 for 100 g day$^{-1}$) and ovarian (RR = 1.53) neoplasms. Weaker trends were found for lung (RR = 1.08) and prostate (RR = 1.19) cancers. For most cancer sites, significant increased risks were found also for the lowest dose of alcohol considered (25 g day$^{-1}$, corresponding to approximately 2 drinks per day). No significant relation emerged between alcohol and pancreas, endometrium and bladder cancers.

Significant heterogeneity across studies was found. Effects of gender in modifying the effect of alcohol intake were investigated for each cancer site, but reached statistical significance only for oesophageal and liver cancers, with higher risks in women for both neoplasms. By pooling the 8 studies reporting the relation between alcohol and the risk of all sites together, significant effects were found starting from intakes of 28 g day$^{-1}$.

To analyse the modifying effect of tobacco, pooled estimates based on unadjusted and adjusted RRs were compared in Table 2 for studies providing relevant information. Effects of smoking adjustment in modifying the effect of alcohol related risks were investigated for 5 neoplasms known to be strongly tobacco-related, but reached statistical significance only for cancers of larynx, lung and bladder, with higher risks for unadjusted estimates. However, while for laryngeal cancer evidence of a substantial alcohol-related risk persisted by pooling studies reporting both unadjusted and adjusted estimates, alcohol did not show significant effects on the risk of lung and bladder neoplasms when its effect was adjusted for smoking. Allowance for tobacco had a negligible effect on the estimates for colorectal cancer. Figure 1 gives the relative risk functions for alcohol consumption, and the corresponding 95% CI of selected neoplasms, by fitting meta-regression models.

### DISCUSSION

This work has some of the limitations, but also most of the strengths of meta-analyses of published studies, including the large number of subjects investigated and the comprehensive picture provided. Its main results are consistent with published meta analyses on alcohol and breast, colorectal (Longnecker et al, 1990; Longnecker, 1994, 1995), bladder (Zeegers et al, 1999) and prostate (Dennis, 2000) cancers.

For most neoplasms considered, estimates tended to be heterogeneous across studies. Consequently, the overall pooled estimates may be systematically influenced by characteristics of the subjects included such as gender, due to the potential differences in alcohol metabolism in women and men (Corrao et al, 1999, 2000). To control for this, whenever possible we included gender term in the meta-regression models. However, gender explained a significant part of the observed heterogeneity only for cancers of oesophagus and liver.

Another open issue is the definition of former drinkers, which in some studies may include only a fraction of former drinkers. However, the time-risk relations between alcohol drinking and cancer risk are complex (Bosetti et al, 2000; Franceschi et al, 2001) and misclassification of former smokers should, if anything, have led to an underestimate of the real association. Further, several cohort studies may have selectively reported relevant findings for selected cancer sites only, and omitted data for less common ones.

It is likely, moreover that alcohol drinking was systematically under-reported in several studies, mostly for selected neoplasms. Consequently, all the RRs would be biased towards lower levels of drinking in case of non-selective under-reporting, or underestimated in the case of selective under-reporting by cases. Specific perplexities concern the pooled estimates for liver cancer, due to pre-existing cirrhosis and consequent reduced alcohol consumption (Aricò et al, 1994; La Vecchia et al, 1998). Further, although this meta-analysis included a total of over 117 000 cases, absolute numbers were relatively limited for some diseases (melanoma, uterus and kidney cancers) or for certain levels of alcohol drinking (high levels for breast cancer in women). Finally, confounding might affect our pooled estimates. However, estimates in the main analysis for upper digestive and respiratory tract cancers, as well as for lung and bladder neoplasms, were adjusted for tobacco, which showed a substantial modifying effect not only for lung and bladder, but also for laryngeal cancer. The moderate excess risk for lung and bladder cancer, moreover, may well be due to some residual confounding by tobacco. No relevant modifying effect was observed for colorectal cancer, which appears to be another tobacco-related neoplasm – though less strongly than the above mentioned ones (D'Avanzo et al, 1995; Giovannucci, 2001).

*© 2001 Cancer Research Campaign* 

**Table 1** Main characteristics of the studies selected for the meta-analysis. Pooled relative risks, and corresponding 95% confidence interval, for selected doses of alcohol consumption are also reported

| | Number | | Study's design | | Pooled RR (and 95% CI) associated with alcohol intake[a] | | | Gender effect (*P*)[b] | Heterogeneity test (*P*)[c] |
|---|---|---|---|---|---|---|---|---|---|
| **Malignancy site** | **Studies** | **Cases** | **Cohort** | **Case–control** | **25 g day⁻¹** | **50 g day⁻¹** | **100 g day⁻¹** | | |
| Oral cavity and pharynx | 26 | 7954 | 1 | 25 | 1.75 (1.70, 1.82) | 2.85 (2.70, 3.04) | 6.01 (5.46, 6.62) | n.s. | < 0.05 |
| Oesophagus | 28 | 7239 | 1 | 27 | 1.51 (1.48, 1.55) | 2.21 (2.11, 2.31) | 4.23 (3.91, 4.59) | < 0.05 | < 0.05 |
| Males | 18 | 3310 | 1 | 17 | 1.43 (1.38, 1.48) | 1.98 (1.87, 2.11) | 3.49 (3.14, 3.89) | – | < 0.05 |
| Females | 5 | 304 | 0 | 5 | 1.52 (1.42, 1.63) | 2.24 (1.95, 2.58) | 4.45 (3.37, 5.87) | | |
| Stomach | 16 | 4518 | 2 | 14 | 1.07 (1.04, 1.10) | 1.15 (1.09, 1.22) | 1.32 (1.18, 1.49) | n.s. | < 0.05 |
| Small intestine | 2 | 415 | 0 | 2 | 1.02 (0.89, 1.17) | 1.04 (0.79, 1.37) | 1.08 (0.63, 1.88) | n.s. | n.s. |
| Colon and rectum | 22 | 11 296 | 6 | 16 | 1.08 (1.06, 1.10) | 1.18 (1.14, 1.22) | 1.38 (1.29, 1.49) | n.s. | < 0.05 |
| Liver | 20 | 2294 | 3 | 17 | 1.17 (1.11, 1.23) | 1.36 (1.23, 1.51) | 1.86 (1.53, 2.27) | < 0.05 | < 0.05 |
| Males | 10 | 949 | 2 | 8 | 1.28 (1.13, 1.45) | 1.51 (1.27, 2.10) | 1.62 (1.18, 2.24) | – | < 0.05 |
| Females | 3 | 231 | 1 | 2 | 1.97 (1.30, 3.00) | 3.57 (1.56, 8.21) | 9.15 (1.73, 48.41) | | |
| Gallbladder | 2 | 81 | 1 | 1 | 1.17 (0.73, 1.86) | 1.36 (0.54, 3.44) | [d] | n.s. | n.s. |
| Pancreas | 17 | 2524 | 4 | 13 | 0.98 (0.90, 1.05) | 1.05 (0.93, 1.18) | 1.18 (0.94, 1.49) | n.s. | < 0.05 |
| Larynx | 20 | 3759 | 0 | 20 | 1.38 (1.32, 1.45) | 1.94 (1.78, 2.11) | 3.95 (3.43, 4.57) | n.s. | < 0.05 |
| Lung | 6 | 2314 | 3 | 3 | 1.02 (1.00, 1.04) | 1.04 (1.00, 1.08) | 1.08 (1.00, 1.18) | n.s. | < 0.05 |
| Melanoma | 2 | 708 | 0 | 2 | 0.50 (0.21, 1.10) | [d] | [d] | n.s. | n.s. |
| Breast | 49 | 44 033 | 12 | 37 | 1.31 (1.27, 1.36) | 1.67 (1.56, 1.78) | 2.71 (2.33, 3.08) | – | < 0.05 |
| Cervix | 1 | 242 | – | 1 | 0.80 (0.50, 1.27) | 0.64 (0.25, 1.60) | – | – | 0.53 |
| Endometrium | 6 | 2473 | 2 | 4 | 1.05 (0.88, 1.24) | 1.09 (0.78, 1.54) | 1.20 (0.60, 2.37) | – | < 0.01 |
| Ovary | 5 | 1651 | – | 5 | 1.11 (1.00, 1.24) | 1.23 (1.01, 1.54) | 1.53 (1.03, 2.32) | – | n.s. |
| Prostate | 11 | 4094 | 4 | 7 | 1.05 (1.00, 1.08) | 1.09 (1.02, 1.17) | 1.19 (1.03, 1.37) | – | n.s. |
| Bladder | 11 | 5997 | 2 | 9 | 1.04 (0.99, 1.09) | 1.08 (0.98, 1.19) | 1.17 (0.97, 1.41) | n.s. | n.s. |
| Kidney | 2 | 921 | 0 | 2 | 0.88 (0.77, 1.02) | 0.79 (0.60, 1.03) | 0.62 (0.36, 1.06) | n.s. | n.s. |
| All sites together | 8 | 14 495 | 6 | 2 | 1.01 (0.90, 1.05) | 1.22 (1.11, 1.27) | 1.91 (1.77, 2.06) | n.s. | < 0.05 |
| Total | 235 | 117 471 | 48 | 187 | – | – | – | – | – |

[a]Pooled relative risk, and corresponding 95% confidence interval (CI), based on multivariate estimates, directly obtained from the β coefficients of the best fitting model, and from the corresponding standard error, respectively (see Methods section); Evidence of a relative risk significantly different from 1 based on 95% CI that does not contain unity ($P < 0.05$). [b]Evidence of significant effect of gender in modifying the effect of alcohol based on significance of the interaction term ($P < 0.05$). [c]Significance ($P < 0.05$) indicates heterogeneity of the effects of alcohol among studies. [d]No studies reported effect of alcohol at the specific dose.

© 2001 Cancer Research Campaign

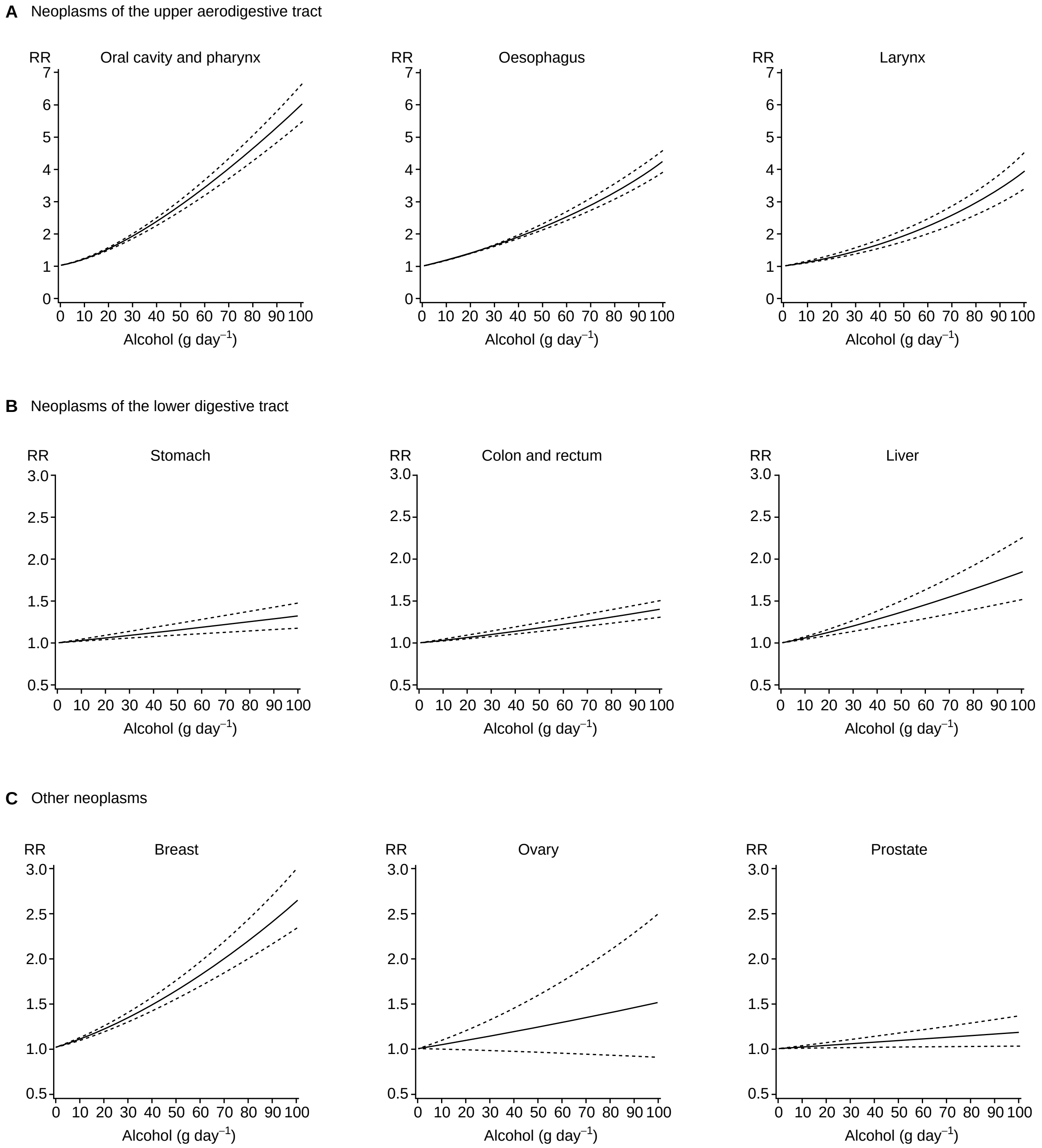

**Figure 1** Relative risk functions, and corresponding 95% confidence intervals, describing the dose–response relationship between alcohol consumption and the risk of the 9 neoplasms showing statistical evidence of alcohol effect

The results were generally inconsistent for cancer of the pancreas, stomach and colorectum, and somewhat inconsistent across studies, possibly following differences in their design. There is also no clear understanding on the possible underlying mechanisms through which alcohol may act as a co-carcinogen on these sites (IARC, 1988; Doll et al, 1999). More importantly, the pooled RR estimates were of the order of 1.1–1.3 for high level of alcohol intake, and residual bias and confounding by diet or other factors is plausible. Inference on a potential causal relationship for these cancer sites is therefore not possible.

The present meta-analysis confirms the existence of a strong dose–risk relation between alcohol and breast cancer risk (Longnecker, 1994). For breast and other hormone-related neoplasm (ovary, endometrium), a role of alcohol on female hormone metabolism and serum levels is plausible (Longnecker, 1994; Dorgan et al, 2001).

*© 2001 Cancer Research Campaign* 

**Table 2** Effect of smoking adjustment in modifying the effect of alcohol on the risk of 5 tobacco-related neoplasms

| Malignancy site | Pooled RR (and 95% CI) associated with alcohol intake[a] | | | Smoking adjustment effect (*P*)[b] | Heterogeneity test (*P*)[c] |
|---|---|---|---|---|---|
| | 25 g day $^{-1}$ | 50 g day $^{-1}$ | 100 g day $^{-1}$ | | |
| Oral cavity | | | | n.s. | < 0.05 |
| Unadjusted estimates | 1.74 (1.67, 1.81) | 2.80 (2.59, 3.04) | 5.82 (5.00, 6.77) | | |
| Adjusted estimates | 1.76 (1.69, 1.82) | 2.87 (2.68, 3.08) | 6.10 (5.45, 6.83) | | |
| Oesophagus | | | | n.s. | < 0.05 |
| Unadjusted estimates | 1.50 (1.47, 1.55) | 2.19 (2.08, 2.31) | 4.18 (3.79, 4.60) | | |
| Adjusted estimates | 1.52 (1.46, 1.57) | 2.23 (2.09, 2.38) | 4.31 (3.84, 4.85) | | |
| Larynx | | | | < 0.05 | < 0.05 |
| Unadjusted estimates | 1.65 (1.55, 1.76) | 2.74 (2.43, 3.09) | 7.45 (6.04, 9.18) | | |
| Adjusted estimates | 1.29 (1.23, 1.36) | 1.68 (1.53, 1.84) | 2.79 (2.36, 3.30) | | |
| Lung | | | | < 0.05 | < 0.05 |
| Unadjusted estimates | 1.58 (1.12, 2.24) | 2.50 (1.25, 5.01) | 6.30 (1.57, 25.18) | | |
| Adjusted estimates | 1.01 (0.99, 1.04) | 1.03 (0.99, 1.08) | 1.07 (0.98, 1.17) | | |
| Bladder | | | | < 0.05 | < 0.05 |
| Unadjusted estimates | 1.16 (1.02, 1.33) | 1.36 (1.04, 1.77) | 1.85 (1.09, 3.13) | | |
| Adjusted estimates | 1.02 (0.97, 1.07) | 1.04 (0.94, 1.15) | 1.09 (0.89, 1.33) | | |

[a]Pooled relative risk, and corresponding 95% confidence interval (CI), directly obtained from the β coefficients of the best fitting model, and from the corresponding standard error, respectively (see Methods section); Evidence of a relative risk significantly different from 1 based on 95% CI that does not contain unity ($P < 0.05$). [b]Evidence of significant effect of smoking adjustment in modifying the effect of alcohol based on significance of the interaction term ($P < 0.05$). [c]Significance ($P < 0.05$) indicates heterogeneity of the effects of alcohol among studies.

Alcohol and tobacco interact in a multiplicative way on the risk of cancers of the upper digestive tract. From a public health viewpoint, such a synergism implies that over 75% of cancers of the upper digestive and respiratory tract in developed countries are attributable to alcohol and tobacco (Negri et al, 1992, 1993; Tavani et al, 1996). While the evidence is inconsistent for laryngeal cancer, the adjusted pooled estimates confirm that for oral and pharyngeal (Talamini et al, 1998; Fioretti et al, 1999) and oesophageal cancers (Doll et al, 1999) alcohol drinking has an independent effect, and hence allowance for tobacco only marginally modified the RR.

Several attempts have been made to separate the effects of different types of alcoholic beverages. Some authors reported no apparent differences, while others have reported greater risks with spirits than with wine or beer (Tuyns et al, 1979; Doll et al, 1999). Whereas a study from Denmark showed no excess risk from wine (Gronbaek et al, 1998), 2 studies from Italy (Barra et al, 1990; Bosetti et al, 2000) found greater risks of oral and pharyngeal and oesophageal cancer in wine drinkers, after adjustment for amount drunk. It appears, therefore, that the most frequently consumed beverage in each area tends to be the one with the highest association (Franceschi et al, 1990). For this reason, we have considered only total alcohol consumption in the present overview.

Notwithstanding the limitations discussed above, this meta-analysis still includes most published information on alcohol and cancer, and consequently – in the absence of a collaborative re-analysis of original data with separation of abstainers and ex-drinkers, and the inclusion of data for the whole range of cancers in cohort studies – provides the most accurate relative risk estimates for most common neoplasms that is available. Some of the findings from this meta-analysis are innovative and of specific relevance, including the absence of a threshold effect for any of major alcohol-related cancer sites, and the apparently stronger association for oral and pharyngeal cancer than for any other site across different levels of alcohol drinking.

### ACKNOWLEDGEMENTS

Supports for this study came from the Italian Ministry of Health, from the Italian Ministry of the University and Scientific and Technologic Research, and the Associazione Italiana per la Ricerca sul Cancro. Ms Ivana Garimoldi provided editorial assistance.

### REFERENCES

Aricò S, Corrao G, Torchio P, Galatola G, Tabone M, Valenti M and Di Orio F (1994) A strong negative association between alcohol consumption and the risk of hepatocellular carcinoma in cirrhotic patients: a case–control study. *Eur J Epidemiol* **10**: 251–257

Barra S, Franceschi S, Negri E, Talamini R and La Vecchia C (1990) Type of alcoholic beverage and cancer of the oral cavity, pharynx and oesophagus in an Italian area with high wine consumption. *Int J Cancer* **46**: 1017–1020

Berlin JA, Longnecker MP and Greenland S (1993) Meta-analysis of epidemiologic dose–response data. *Epidemiology* **4**: 218–228

Bosetti C, Franceschi S, Levi F, Negri E, Talamini R and La Vecchia C (2000a) Smoking and drinking cessation and the risk of oesophageal cancer. *Br J Cancer* **83**: 689–691

Bosetti C, La Vecchia C, Negri E and Franceschi S (2000b) Wine and other types of alcoholic beverages and the risk of esophageal cancer. *Eur J Clin Nutr* **54**: 918–920

Corrao G, Bagnardi V, Zambon A and Aricò S (1999) Exploring the dose–response relationship between alcohol consumption and the risk of several alcohol-related conditions: a meta-analysis. *Addiction* **94**: 1551–1573

Corrao G, Rubbiati L, Bagnardi V, Zambon A and Poikolainen K (2000) Alcohol and coronary heart disease: a meta-analysis. *Addiction* **95**: 1505–1523

D'Avanzo B, La Vecchia C, Franceschi S, Gallotti L and Talamini R (1995) Cigarette smoking and colorectal cancer: a study of 1,584 cases and 2,879 controls. *Prev Med* **24**: 571–579

Dennis L (2000) Meta-analysis for combining relative risks of alcohol consumption and prostate cancer. *Prostate* **42**: 56–66

Doll R, Forman D, La Vecchia C and Woutersen R (1999) Alcoholic beverages and cancers of the digestive tract and larynx. In: Health Issue related to Alcohol Consumption, Macdonald I (ed) pp 351–393. 2nd ed. Oxford: ILSI Europe, Blackwell Science Ltd

 *© 2001 Cancer Research Campaign*

Dorgan JF, Baer DJ, Albert PS, Judd JT, Brown ED, Corle DK, Campbell WS, Hartman TJ, Tejpar AA, Clevidence BA, Giffen CA, Chandler DW, Stanczyk FZ and Taylor PR (2001) Serum hormones and the alcohol-breast cancer association in postmenopausal women. *J Natl Cancer Inst* **93**: 710–715

Fioretti F, Bosetti C, Tavani A, Franceschi S and La Vecchia C (1999) Risk factors for oral and pharyngeal cancer in never smokers. *Oral Oncol* **35**: 375–378

Franceschi S, Talamini R, Barra S, Baròn AE, Negri E, Bidoli E, Serraino D and La Vecchia C (1990) Smoking and drinking in relation to cancers of the oral cavity, pharynx, larynx, and esophagus in northern Italy. *Cancer Res* **50**: 6502–6507

Franceschi S, Levi F, Dal Maso L, Talamini R, Conti E, Negri E and La Vecchia C (2000) Cessation of alcohol drinking and risk of cancer of the oral cavity and pharynx. *Int J Cancer* **85**: 787–790

Giovannucci E (2001) An updated review of the epidemiological evidence that cigarette smoking increases risk of colorectal cancer. *Cancer Epidemiol Biomarkers Prev* **10**: 725–731

Greenland S and Longnecker MP (1992) Methods for trend estimation from summarized dose-response data, with applications to meta-analysis. *Am J Epidemiol* **135**: 1301–1309

Gronbaek M, Becker U, Johansen D, Tonnesen H, Jensen G and Sorensen TI (1998) A population-based cohort study of the association between alcohol intake and cancer of the upper digestive tract. *BMJ* **317**: 844–848

IARC (1988) IARC Monographs on the Evaluation of Carcinogenic Risks to Humans. Vol. 44, Alcohol Drinking. *Lyon IARC* pp 416

La Vecchia C, Negri E, Cavalieri D'Oro L and Franceschi S (1998) Liver cirrhosis and the risk of primary liver cancer. *Eur J Cancer Prev* **7**: 315–320

Longnecker MP (1994) Alcoholic beverage consumption in relation to risk of breast cancer: meta-analysis and review. *Cancer Causes Control* **5**: 73–82

Longnecker MP (1995) Alcohol consumption and risk of cancer in humans: an overview. *Alcohol* **12**: 87–96

Longnecker MP and Enger SM (1996) Epidemiologic data on alcohol beverage consumption and risk of cancer. *Clin Chim Acta* **246**: 121–141

Longnecker MP, Orza MJ, Adams ME, Vioque J and Chalmers TC (1990) A meta-analysis of alcoholic beverage consumption in relation to risk of colorectal cancer. *Cancer Causes Control* **1**: 59–68

Negri E, La Vecchia C, Franceschi S, Decarli A and Bruzzi P (1992) Attributable risks for oesophageal cancer in Northern Italy. *Eur J Cancer* **28A**: 1167–1171

Negri E, La Vecchia C, Franceschi S and Tavani A (1993) Attributable risk for oral cancer in Northern Italy. *Cancer Epidemiol Biomarkers Prev* **2**: 189–193

Royston P, Ambler G and Sauerbrei W (1999) The use of fractional polynomials to model continuous risk variables in epidemiology. *Int J Epidemiol* **28**: 964–974

Talamini R, La Vecchia C, Levi F, Conti E, Favero A and Franceschi S (1998) Cancer of the oral cavity and pharynx in nonsmokers who drink alcohol and in nondrinkers who smoke tobacco. *J Natl Cancer Inst* **90**: 1901–1903

Tavani A, Negri E, Franceschi S, Barbone F and La Vecchia C (1994) Attributable risks for laryngeal cancer in Northern Italy. *Cancer Epidemiol Biomarkers Prev* **3**: 121–125

Tuyns AJ, Pequignot G and Abbatucci JS (1979) Oesophageal cancer and alcohol consumption: importance of type of beverage. *Int J Cancer* **23**: 443–447

Zeegers MPA, Tan FES, Verhagen AP, Weijenberg MP and van den Brandt PA (1999) Elevated risk of cancer of the urinary tract for alcohol drinkers: a meta-analysis. *Cancer Causes Control* **10**: 445–451

*© 2001 Cancer Research Campaign*
